# Xpert^®^ MTB/RIF associated with improved treatment initiation among patients with smear-negative tuberculosis

**DOI:** 10.5588/ijtld.17.0460

**Published:** 2018-12-01

**Authors:** S. Zawedde-Muyanja, Y. C. Manabe, N. K. Sewankambo, L. Nakiyingi, D. Nakanjako

**Affiliations:** * The Infectious Diseases Institute, Makerere University College of Health Sciences, Kampala, Uganda; † Department of Medicine, School of Medicine, Makerere University College of Health Sciences, Kampala, Uganda; ‡ Division of Infectious Diseases, Department of Medicine, Johns Hopkins University School of Medicine, Baltimore, Maryland, USA

**Keywords:** tuberculosis, diagnosis, linkage to treatment

## Abstract

**BACKGROUND:**

Delayed diagnosis and treatment initiation of smear-negative tuberculosis (TB) patients can lead to increased morbidity and mortality, particularly among those co-infected with the human immunodeficiency virus (HIV).

**OBJECTIVE:**

To compare TB treatment initiation among smear-negative presumptive TB patients in the 6 months before and after the introduction of Xpert^®^ MTB/RIF testing at five rural tertiary hospitals in Uganda.

**METHODS:**

Patient records of the dates and results of sputum analysis were extracted from TB laboratory registers and linked to those on treatment initiation as indicated in the TB treatment registers. The proportion of smear-negative presumptive patients who initiated anti-tuberculosis treatment was compared before and after Xpert implementation using χ^2^ tests. Time to treatment was analysed using Kaplan-Meier survival analysis.

**RESULTS:**

Records from 3658 patients were analysed, 1894 before and 1764 after the introduction of Xpert testing. After the introduction of Xpert, 25% (437/1764) of smear-negative presumptive TB patients underwent testing. The proportion initiated on anti-tuberculosis treatment increased from 5.9% (112/1894) to 10.8% (190/1764) (*P* < 0.01). However, 37% (32/87) of patients with a confirmed TB diagnosis did not initiate treatment. Time to TB treatment initiation improved from 8 to 3.5 days between the study periods.

**CONCLUSION:**

Xpert testing was associated with improved TB treatment initiation among smear-negative presumptive TB patients. Improved utilisation and linkage to treatment could improve the impact of this test on patient-centred outcomes.

## Abstract (FR)

**CONTEXTE:**

Le retard au diagnostic et à la mise en route du traitement de la tuberculose (TB) à frottis négatif aboutit à une augmentation de la morbidité et de la mortalité, particulièrement parmi les patients coinfectés par le virus de l'immunodéficience humaine (VIH).

**OBJECTIF:**

Comparer la mise en route du traitement de TB parmi des patients présumés TB à frottis négatif dans les 6 mois précédant et suivant l'introduction du test Xpert^®^ MTB/RIF dans cinq hôpitaux tertiaires ruraux d'Ouganda.

**MÉTHODE:**

Les données des patients relatives à la date et aux résultats de l'analyse de crachats ont été extraites du registre du laboratoire TB et liés à ceux de la mise en route du traitement antituberculeux comme le montrent les registres de traitement de la TB. Les analyses univariées des caractéristiques des patients vus dans les hôpitaux ont été décrites en fréquences et en pourcentages. Les caractéristiques des patients associées au fait de bénéficier du test Xpert ont été analysées par régression logistique multivariée. La proportion de patients présumés à frottis négatif qui ont débuté le traitement antituberculeux a été comparée dans les deux périodes par test du χ^2^. Le délai de traitement a été analysé par les courbes de survie de Kaplan-Meier.

**RÉSULTATS:**

Les dossiers de 3658 patients ont été analysés : 1894 avant et 1764 aprés l'introducion de l'Xpert. Après l'introduction du Xpert, 25% (437/1764) des patients présumés TB à frottis négatif ont eu un test. Le sexe masculin, un statut VIH non enregistré et un âge ⩾55 ans ont été significativement associés au fait de ne pas bénéficier d'un test Xpert. La proportion de patients mis sous traitement de TB a augmenté de 5,9% (112/1894) à 10,8% (190/1764) (*P* < 0,01). Cependant, 37% (32/87) des patients ayant un diagnostic de TB confirmé n'ont pas mis en route le traitement. Le délai de démarrage du traitement a diminué de 8 à 3,5 jours entre les deux périodes de l'étude.

**CONCLUSION:**

Le test Xpert a été associé avec une amélioration de la mise en route du traitement de TB parmi des patients présumés TB à frottis négatif. Une meilleure utilisation du test et un lien vers le traitement pourraient améliorer l'impact de ce test sur les résultats centrés sur le patient.

## Abstract (SP)

**MARCO DE REFERENCIA:**

El retraso en el diagnóstico y el inicio del tratamiento de la tuberculosis (TB) con baciloscopia negativa da lugar a un aumento de la morbilidad y la mortalidad, sobre todo en los pacientes coinfectados por el virus de la inmunodeficiencia humana (VIH).

**OBJETIVO:**

Comparar el momento del inicio del tratamiento antituberculoso en los pacientes con presunción de TB y baciloscopia negativa en los 6 meses que precedieron y los 6 meses que siguieron a la introducción de la prueba Xpert^®^ MTB/RIF en cinco hospitales rurales de atención terciaria en Uganda.

**MÉTODOS:**

Los datos de los pacientes sobre las fechas y los resultados del análisis de esputo se extrajeron del registro del laboratorio de TB y se vincularon con los datos de los pacientes que habían iniciado el tratamiento antituberculoso, según los registros de tratamiento de la TB. En los análisis univariantes, las características de los pacientes atendidos en los hospitales se describieron en forma de frecuencias y porcentajes. Las características de los pacientes que se asociaron con el hecho de recibir la prueba Xpert se analizaron mediante una regresión logística multivariante. Se aplicó la prueba de la χ^2^ al comparar la proporción de pacientes con presunción de TB y baciloscopia negativa que iniciaron tratamiento antituberculoso en los dos períodos. El lapso hasta el inicio del tratamiento se analizó con el análisis de Kaplan-Meier.

**RESULTADOS:**

Se analizaron las historias clínicas de 3658 pacientes: 1894 antes y 1764 despuès de la introduccion de la prueba Xpert. Después de la introducción de Xpert, el 25% de los pacientes con presunción de TB y baciloscopia negativa (437/1764) recibió una prueba diagnóstica. Los factores asociados de manera significativa con el hecho de no recibir Xpert fueron el sexo masculino, la falta de registro de la situación frente al VIH y una edad ⩾55 años. La proporción de pacientes que inició el tratamiento antituberculoso aumentó de 5,9% (112/1894) a 10,8% (190/1764) (*P <* 0,01). Sin embargo, el 37% de los pacientes (32/87) con un diagnóstico de TB confirmado no inició tratamiento. El lapso hasta el inicio del tratamiento antituberculoso entre los dos períodos disminuyó de 8 días a 3,5 días.

**CONCLUSIÓN:**

La prueba Xpert se asoció con progresos en el inicio del tratamiento antituberculoso de los pacientes con presunción clínica y baciloscopia negativa. Una mayor utilización del dispositivo y un mejor vínculo con los servicios de tratamiento podría mejorar la repercusión de esta prueba en los desenlaces centrados en el paciente.

TUBERCULOSIS (TB) REMAINS A MAJOR cause of morbidity and mortality among patients with the human immunodeficiency virus (HIV), even in the era of antiretroviral therapy (ART). In 2015, an estimated 1.2 million people who developed TB worldwide were co-infected with HIV and, among HIV-TB co-infected individuals, mortality was more than double that among TB patients without HIV.^1^ In Uganda, almost half of all TB patients treated annually by the National TB and Leprosy Programme (NTLP) are co-infected with HIV.^1^,^2^ In 2014, one third of these patients died during TB treatment,^1^ three times the estimated mortality among HIV-negative TB patients. One of the main contributors to mortality among TB-HIV co-infected patients is delayed TB diagnosis and treatment initiation;^3–6^ this is often a consequence of presentation with smear-negative TB, which is difficult to diagnose.^7^,^8^

In 2011, the Xpert^®^ MTB/RIF assay (Cepheid, Sunnyvale, CA, USA), an automated molecular test with diagnostic sensitivity and specificity comparable with sputum culture,^9^ was approved by the World Health Organization (WHO) for use in resource-limited, high TB burden settings, including Uganda.^10^,^11^ Because of its speed and accuracy of diagnosis, Xpert has the potential to reduce TB-related morbidity and mortality, particularly in smear-negative patients. Xpert testing was subsequently rolled out by the Uganda NTLP in 2012. The test was introduced in a phased manner, starting with Regional Referral Hospitals (RRHs), and was initially recommended for smear-negative presumptive TB patients who were HIV-infected or whose HIV status was not known.^10^ To increase access to the test, the NTLP set up a specimen transportation system to create inter-facility linkages between these hospitals and lower-level health facilities.^12^ In view of the high cost of setting up and maintaining Xpert services^13^,^14^ and to evaluate WHO and country recommendations, it is important to investigate whether Xpert roll-out has resulted in an improvement in patient outcomes, e.g., increased and more rapid TB treatment initiation among presumptive TB patients.

We aimed to assess the effect of the introduction of Xpert on the proportion of smear-negative presumptive TB patients initiated on anti-tuberculosis treatment and on the time to treatment initiation in these patients at selected tertiary hospitals in Uganda. We hypothesised that a more sensitive, rapid tool for TB confirmation would increase the proportion of smear-negative presumptive patients (the majority of whom are HIV-infected) treated for TB and reduce the time to treatment initiation among these patients.

## METHODS

### Study setting

RRHs are tertiary hospitals that, in addition to in-and out-patient care, offer specialist clinical, radiological and surgical services. Each hospital serves 10 districts, a population of about 2 500 000, and has medical out-patient clinics which see 7000–10 000 patients each month. An HIV/ART clinic attached to each medical out-patient clinic serves 3000–5000 active TB patients.^2^ The out-patient clinics are serviced by fully functioning laboratories equipped with fluorescence microscopy (FM) and Xpert.

The results of all sputum samples tested in these laboratories were recorded in the hospitals' TB laboratory registers. TB treatment is offered at all RRHs using internationally recommended fixed-dose regimens provided by the Uganda NTLP, and is recorded in the hospitals' TB treatment register.

**Figure 1.**
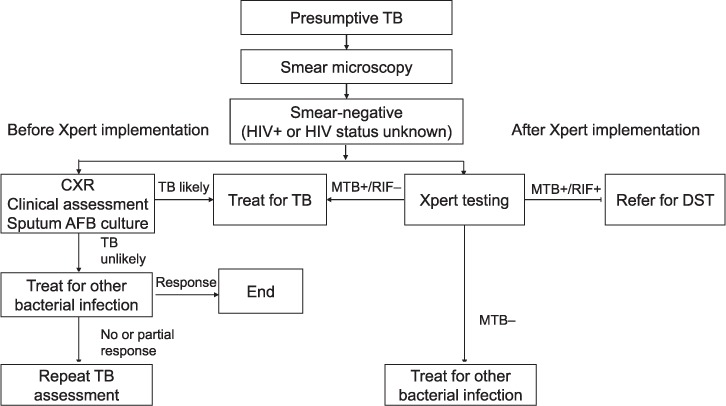
Algorithms for TB diagnosis in Uganda before and after implementation of Xpert^®^ MTB/RIF testing. TB=tuberculosis; HIV=human immunodeficiency virus; +=positive; CXR=chest X-ray; AFB = acid-fast bacilli; MTB = M. tuberculosis; RIF = rifampicin; −= negative; DST = drug susceptibility testing.

From January 2012 to December 2014, Xpert testing was sequentially introduced at all RRHs in Uganda. Five of these hospitals were included in the study based on the availability and completeness of data 6 months before and 6 months after the introduction of Xpert. These hospitals serve large rural populations in Eastern, Northern and Western Uganda. Before the introduction of Xpert, presumptive TB patients with a smear-negative result were started on anti-tuberculosis treatment if they 1) had a chest X-ray suggestive of TB, 2) did not respond to a 2-week course of antibiotics, or 3) if the health care worker made a clinical decision to start them on anti-tuberculosis treatment (Figure 1). After the introduction of Xpert, presumptive TB patients with a negative smear result were recommended for Xpert testing if they were HIV-positive or their HIV status was not known. Patients with a positive Xpert test were started on treatment for drug-susceptible TB if their disease was rifampicin (RMP) susceptible or, if their disease was RMP-indeterminate or -resistant they were referred for drug susceptibility testing and subsequent treatment for drug-resistant TB. Patients who were negative on Xpert were referred back to health care workers to be assessed and treated for another possible cause of their symptoms (Figure 1).

### Study subjects, procedures and measurements

Patient records were retrieved from the hospital's TB laboratory register 6 months before and 6 months after the introduction of Xpert. Records from presumptive TB patients who were aged ⩾15 years, had at least one negative smear result and were HIV-positive or had unknown HIV status were included in our study. Records from patients whose HIV status was recorded as negative were not included in the study because, for the period covered by this analysis, Xpert was not recommended for HIV-negative patients. Records were excluded if patients' second smear result was positive or if they were diagnosed with RMP-resistant TB. Data on dates of sputum examination, dates of Xpert testing and the results of these tests were recorded. The data were then linked to results from the hospital TB treatment registers using a manual linkage system based on four parameters: the patient's name, age and sex and place of residence. From the hospital treatment registers, data on the date of TB treatment initiation, type of TB regimen and ART initiation (if the patient was co-infected with HIV) were extracted. For patients diagnosed at the tertiary hospital but started on treatment at a lower-level health facility, data on treatment initiation were obtained from the district TB register.

### Study definitions

For this study, a presumptive TB patient was described as a patient with two or more (one or more if HIV-positive) of the following TB symptoms: 1) cough for >2 weeks (cough of any duration in those who were HIV-positive), 2) persistent fevers, 3) night sweats, or 4) unexplained weight loss, as described in the WHO intensified case finding guide.^15^ Patients were diagnosed with TB if they had a positive smear or Xpert result recorded in the hospital's laboratory TB register or if they were smear or Xpert negative and a decision was made by a clinician to start them on anti-tuberculosis treatment.

Patients were initiated on treatment if they had anti-tuberculosis treatment with an internationally recommended, fixed-dose TB regimen that was recorded in the hospital's TB treatment register.

Time to treatment initiation was defined as the number of days between the first smear test result and the start of anti-tuberculosis treatment.

### Statistical analysis

Univariate analyses of the characteristics of the patients seen at the hospitals and those undergoing Xpert were described using frequencies and percentages. Multivariate logistic regression was used to identify the patient characteristics associated with undergoing Xpert. The proportion of smear-negative presumptive patients initiated on treatment was compared for the period before and after Xpert implementation using χ^2^ tests. The time to TB treatment initiation for the period before and after Xpert implementation was analysed using Kaplan-Meier survival analysis curves. The survival distributions of the two time periods were compared using the log-rank test.

### Ethics statement

The study protocol was approved by the Mengo Hospital Research and Ethics Review Board, Kampala (787/10–15), and by the Uganda National Council of Science and Technology, Kampala, Uganda (HS 1998).

## RESULTS

**Figure 2.**
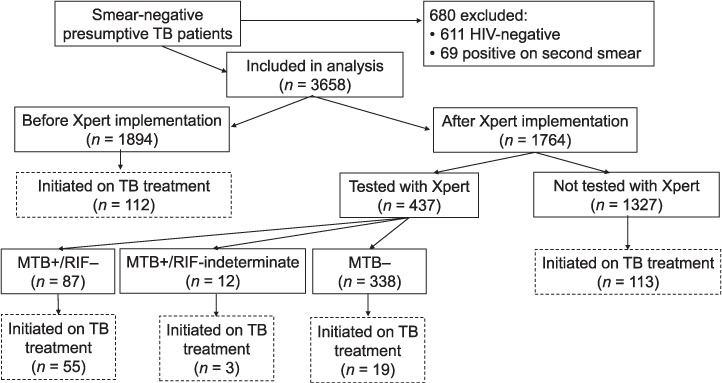
Flow chart of adult smear-negative TB patients examined at five tertiary hospitals 6 months before and after the implementation of Xpert^®^ MTB/RIF testing. TB =tuberculosis; HIV = human immunodeficiency virus; MTB=M. tuberculosis;+=positive; RIF=rifampicin;−=negative.

During the study period, records from 4338 presumptive TB patients with an initial smear-negative result were retrieved from the TB laboratory registers of participating hospitals. Of these, 680 were excluded from analyses because they had a positive result on their second smear (*n* = 69) or their HIV status was recorded as negative (*n* = 611). Records from 3658 patients were analysed: 1894 patients before and 1764 patients after the introduction of Xpert testing. Of these, 302 were initiated on anti-tuberculosis treatment: 5.9% (112/1894) patients before and 10.8% (190/1764) patients after the introduction of Xpert testing (Figure 2).

The baseline characteristics of presumptive TB patients in both time periods were similar, except for the proportion of presumptive TB patients who had a documented HIV-positive result, which was higher after the implementation of Xpert, probably due to improved HIV testing among presumptive TB patients during this period (Table 1). In both periods, one third of all presumptive TB patients did not return to the health facility for a second smear test.

**Table 1:**
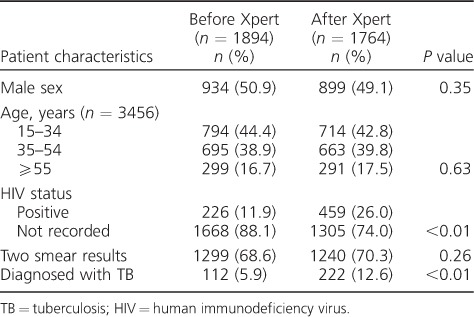
Baseline characteristics of smear-negative presumptive TB patients

Overall, utilisation of Xpert was low at all hospitals, with an average of only 25% of smear-negative presumptive TB patients accessing the test (Figure 2). In multivariate logistic regression analysis, male sex, having an unrecorded HIV status and age ⩾55 years were significantly associated with not undergoing Xpert (Table 2). The proportion of presumptive TB patients tested with Xpert ranged from 10% to 54% and, with the exception of one hospital with very high rates of empirical treatment, was higher at hospitals that had a higher proportion of smear-negative presumptive patients diagnosed with TB (Table 3).

**Table 2:**
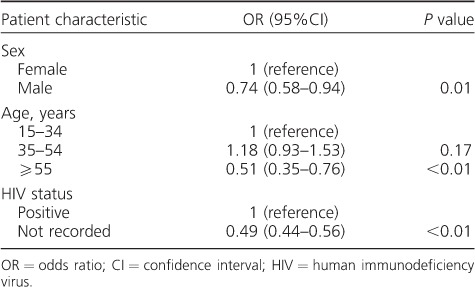
Results of multivariate analysis of patient characteristics associated with undergoing Xpert

**Table 3:**
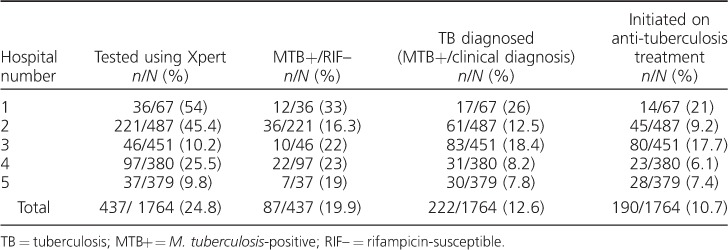
Smear-negative presumptive TB patients examined using Xpert, diagnosed with TB and initiated on anti-tuberculosis treatment at five regional referral hospitals in Uganda

The proportion of smear-negative presumptive TB patients initiated on anti-tuberculosis treatment increased from 5.9% (112/1894) before the introduction of Xpert to 10.8% (190/1764) (*P* < 0.01) after. However, the proportion of presumptive patients initiated on anti-tuberculosis treatment was lower than the proportion with a confirmed TB diagnosis, due to inadequate linkage to treatment of patients diagnosed with TB (Table 2). Overall, 32/87 (37%) Xpert-positive, RMP-susceptible patients did not initiate treatment (Figure 2).

**Figure 3.**
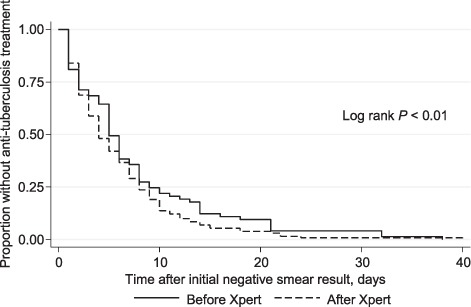
Kaplan-Meier curves for time to treatment initiation in smear-negative tuberculosis patients stratified by the availability of Xpert testing.

Anti-tuberculosis treatment was initiated more rapidly among patients with smear-negative TB after the introduction of Xpert. The mean time to treatment was reduced from 8 days before the introduction of Xpert to 3.5 days after. Survival analysis curves showed a statistically significant reduction in time to TB treatment initiation after the introduction of Xpert (Figure 3).

## DISCUSSION

This retrospective study showed low utilisation of Xpert in the initial months of implementation, with only 25% of all eligible patients undergoing Xpert. Low utilisation of Xpert in the initial months of implementation has been demonstrated by another study from Uganda, in which only 21% of all smear-negative presumptive TB patients underwent Xpert,^17^ and in other high-burden settings such as Swaziland and Malawi, where 50% and 33% of eligible patients received Xpert.^17^,^18^ In our setting, low utilisation of Xpert could be attributed to high levels of empirical treatment, which has been demonstrated among health care workers in tertiary care settings in Uganda.^19^ In the present study, 60% of all smear-negative presumptive TB patients initiated on anti-tuberculosis treatment after the introduction of Xpert were treated empirically. Male sex, age ⩾55 years and having an unrecorded HIV status were significantly associated with not receiving Xpert. Other studies have shown that male sex and older age are associated with reduced access to TB diagnostic services. Findings from countrywide TB prevalence surveys, including the one conducted in Uganda in 2015, have revealed high numbers of previously undiagnosed TB patients among these patient groups.^20–22^ In our study, older patients may have found it difficult to find the means to return to hospital for another sputum examination, while for most men who were wage earners, long waiting times at these hospitals may have been a deterrent to returning for a second sputum examination.

In our study, the implementation of Xpert was associated with an increase in TB treatment initiation among smear-negative presumptive TB patients. These findings are similar to observations from studies carried out at tertiary hospitals in Uganda and India, where the introduction of Xpert increased the proportion of smear-negative presumptive TB patients started on treatment by respectively 10% and 28%.^21^,^22^ Our findings are different from another programmatic evaluation of the impact of Xpert in Uganda that showed no impact of Xpert testing on the proportion of smear-negative patients started on treatment.^16^ In the latter study, the proportion of smear-negative presumptive TB patients examined with Xpert was slightly lower, at 21%, and was comparable with the proportion at hospitals that showed no impact of Xpert testing in our study.

The impact of Xpert on the number of patients initiated on treatment was weakened by inadequate linkage to anti-tuberculosis treatment for patients diagnosed with TB: 37% of Xpert-positive patients were not initiated on anti-tuberculosis treatment. This scenario has been reported in studies from other high-burden countries such as South Africa and Mozambique, where respectively 24% and 33% of patients diagnosed with TB using Xpert testing were not started on treatment.^23^,^24^ In those studies, the failure to link to anti-tuberculosis treatment was mainly due to difficulties in relaying sputum results back to peripheral health facilities from central laboratories. Although our study only included patients evaluated at the Xpert testing site, our findings highlight weaknesses within the health care system and the need to improve TB care processes to ensure linkage to treatment for all patients diagnosed with TB, even at health facilities where Xpert testing is located.

Similar to findings from South Africa and Brazil, Xpert was associated with reduced time to TB treatment initiation.^25^,^26^ Although our study did not assess the impact of this improved time to treatment initiation on mortality from TB, other studies have shown that, despite more rapid treatment initiation, Xpert testing has not had an impact on patient mortality.^22^ This may be due to late presentation of TB patients to health facilities. In Uganda, the time between symptom onset and presentation to a health facility can be as long as 3 months.^29^

Our study had two main limitations. First, as this is a quasi-experimental pre-post study, the observed increase in TB treatment initiation could be attributed to other improvements in health care delivery, such as the training of health care workers or improved record-keeping between the two time periods. This limitation was minimised by keeping the period of analysis short. Second, the study data collected under routine programmatic conditions resulted in the exclusion of health facilities with incomplete patient records, which may have introduced bias into the study. However, despite these limitations, the study has shown the impact of the benefits of Xpert in reducing treatment initiation delays among smear-negative patients and highlighted improvements that could be made in health care delivery to achieve greater benefits from this test.

## CONCLUSION

Xpert testing was associated with improvements in TB treatment initiation among smear-negative presumptive TB patients. Suboptimal utilisation and inadequate linkage to anti-tuberculosis treatment reduced the impact of Xpert testing. Health systems strengthening approaches that focus on improving utilisation and linkage to treatment for patients diagnosed with TB could improve the impact of this test on patient-centred outcomes.
